# Medical Education

**Published:** 1884-11

**Authors:** 


### Medical Education.

At the annual meetings of the various medical organizations, State and National, which have been held during the past three months, the subject of preliminary education of medical students has received more than usual attention. Almost unanimously, the individual members of the profession, and the various organizations as units, (with one single exception,) have pronounced in favor of exacting proof of proper preliminary education before admitting candidates to the lecture classes. There is, practically, no opposition to the movement, the only dissentients now remaining being a few members of college faculties, influenced probably, by a fear of diminishing classes. With a few exceptions, and these diminishing in number from time to time, the better class of colleges have already adopted this requirement. Every announcement for the session of 1884–85 thus far received makes this a distinctive requirement, but it is to be wished that the colleges would state specifically in their announcements the kind of examinations applicants would be subjected to, or the proof of fitting education required, instead of merely saying, as many of them do, "a preliminary education is required."

As illustrating the widespread influence of the effort to heighten the standard of professional acquirements, it may be stated that at a recent meeting of the Nebraska State Medical Society the qualifications for admission to membership were so amended as to require that applicants must be graduates of colleges which in all respects conform to the standard of minimum requirements of this board.

